# Seroprevalence of human immunodeficiency virus, hepatitis B and C viruses, and *Treponema pallidum* infections among blood donors at Shiyan, Central China

**DOI:** 10.1186/s12879-016-1845-z

**Published:** 2016-10-01

**Authors:** Shuguo Yang, Danmei Jiao, Changjun Liu, Ming Lv, Shan Li, Zongyun Chen, Yao Deng, Yanqing Zhao, Jian Li

**Affiliations:** 1 Institute of Basic Medical Science, College of Basic Medicine; Department of Infectious Disease, Renmin Hospital, Hubei University of Medicine, Shiyan, 442000 China; 2 Shiyan Blood Transfusion Center, Laboratory Medical Center, Shiyan, 442000 China; 3 Department of Preventive Medicine, College of Public Health and Management, Hubei University of Medicine, Shiyan, 442000 China; 4 Jiangsu Institute of Parasitic Diseases, Wuxi, 214064 China

**Keywords:** Seroprevalence, Transfusion-transmissible infections, Shiyan, Risk factors

## Abstract

**Background:**

Ordinary screening of transfusion-transmissible infections (TTIs) among blood donors is essential for blood transfusion. Although there is several TTIs studies focus on human immunodeficiency virus, hepatitis B and C viruses, and *Treponema pallidum* infections in China, it is no data to illustrate any firm conclusion from Shiyan City, Central China. It aims to verify the seroprevalence of TTIs among blood donors at Shiyan.

**Methods:**

A retrospective analysis of blood donors’ information was conducted for the presence of HIV, HBV, HCV and *T. pallidum*. Logistic regression analysis was used to demonstrate risk factors including age, gender and occupation associated with them. The variation tendency in seroprevalence of these TTIs over the study period was evaluated by Cochran-Armitage trend test.

**Results:**

Of 211 639 blood donors, 2 858 (1.35 %) had serological evidence of TTIs. The seroprevalence of HIV, HBV, HCV and *T. pallidum* were 0.08 %, 0.51 %, 0.20 % and 0.57 %, respectively. However, the co-infection prevalence of TTIs has not been detected. The HIV seropositivity significantly increased among female donors (OR = 1.63, *P* < 0.001) and farmers (OR = 2.02, *P* = 0.020). Significantly increased HBV seropositivity was only observed framers (OR = 1.87, *P* <0.001) compared to workers. Analogously, significantly increased HCV seropositivity was observed among farmers (OR = 2.59, *P* < 0.001), students (OR = 2.43, *P* < 0.001), merchants (OR = 1.70, *P* = 0.014) and others (OR = 1.78, *P* =0.001). The *T. pallidum* seroprevalence was notably increased among female (OR = 1.54, *P* < 0.001), and farmers (OR = 1.70, *P* <0.001). Moreover, significantly increasing trends of HIV (Z = −6.88, *P* < 0.01), HBV (Z = −4.52, *P* < 0.01), HCV (Z = −4.16, *P* < 0.01) and *T. pallidum* (Z = −1.36, *P* < 0.01) seropositivity were observed over the study period.

**Conclusions:**

It originally offers a substantial prevalence of TTIs among blood donors at Shiyan, Central China. Severe blood donor selection and all-inclusive screening of blood are highly recommended. It might be helpful for developing and updating guidance for blood safety.

**Trial registration:**

Retrospectively registered.

## Background

Transfusion-associated infections, mainly human immunodeficiency virus (HIV), hepatitis B virus (HBV), hepatitis C virus (HCV), and *Treponema pallidum* among blood donors are serious public health problems. The World Health Organization (WHO) recommends conventional and quality-controlled screening of blood donations for the major TTIs. Over three decades, the threats of TTIs have been radically dropped via normal blood testing of blood-borne pathogens [1–3].

While the transfusion-transmissible diseases, which including Acquired Immune Deficiency Syndrome (AIDS) caused by HIV and viral hepatitis infected by HBV, HCV, proceed to spread particularly in less developed countries and areas. In 2015, China has harboured 18.84 % of the world population. Thus, there is no doubt that the blood supply in China has the potential to influence the global blood transfusion. Last several decades, all levels Blood Transfusion Station and Center in China has strengthened the management of blood examination and supply. Although the significant achievements has already obtained during the process, overwhelming problems remain exists [4]. In China, high prevalence of TTIs including HIV, HBV, HCV and *T. pallidum* in the general populations poses an enormous hazard to blood safety [5]. Despite a series of studies have surveyed the TTIs in China [5–8], data revealing the TTIs epidemiology among blood donors is drastically limited. In addition, accompanied the accelerated social transformation and population mobility, infectious diseases profile transfers from intestinal infectious diseases to bloodborne diseases and sexually transmitted diseases (STDs).

In Central China, a car city named Shiyan, which is also the water source of the Middle Route Project for South to North Water Transfer, has 3 million populations including 1 million permanent resident populations and 2 million floating populations. In partial medical institutions at different levels (3 of class III comprehensive hospital, 2 of Tertiary specialized hospital, 15 of the secondary and the following medical institutions), the blood consumption was increased from 70 thousand units to 113 thousand units between 2008 and 2010 [9]. Thus, the blood safety is significant essential and should be paid enough attention. However, Bloodborne Infectious Diseases (HIV/AIDS, Hepatitis B & C) and STDs in the area have not been reported. The main purpose of current study was to understand the seroprevalence of pathogens including HIV, HBV, HCV, and *T. pallidum* from blood donors between 2010 and 2014 at Shiyan city, which firstly allowed a sight of increasing incidence of TTIs in this city of China. The blood safety can be anticipated via monitoring the prevalence of bloodborne pathogens from blood donations, and then a dependable indicator for policy formulation can be measured.

## Methods

### Study design, setting and subjects

A retrospective analysis of data from consecutive, voluntary blood donors between January 2010 and December 2014 was carried out at Shiyan Blood Transfusion Center (SYBTC) and Hubei University of Medicine. Before the screening, the potential blood donors’ medical history was checked. Subjects are required to answer questions related to previous illnesses and medical conditions. History of blood transfusion and questions interrelated to unsafe sex are also involved. Healthy individuals (Age 18 to 65 years) with body weight ≥45 kg would meet the criteria for blood donation. For healthy blood donors, the medical and socio-demographic information were recorded and venous blood were collected in blood banking bags following Standard Operation Procedures (SOPs).

### Ethical statement

All studies were approved by the Ethics Committee of Shiyan Blood Transfusion Center and Hubei University of Medicine. Nevertheless, because of the survey and its limitations (retrospective review of blood donors’ records), informed consent was not gained from the study individuals. Then, the gathered information of individuals was anonymized and de-identified prior to analysis.

### ABO blood grouping and Rhesus (RH) typing

Determinations of ABO blood groups were carried out on a microslide using monoclonal blood grouping antisera: anti-A, anti-B, and anti-AB (Shanghai Hemo-Pharmaceutical and Biological Co., Ltd., Beijing, China). Rh blood groups determinations were examined on a microslide with monoclonal blood grouping anti-D with Anti-D Blood Grouping Reagent (Millipore UK Ltd., Livingston, United Kingdom).

### Immunoanalysis of HIV, HBV, HCV and *T. pallidum*

Serum sample of each blood donor was screened for HIV using Anti-HIV kit (Beijing Wantai Biological Pharmacy Enterprise Co., Ltd., Beijing, China) and HIV Ag/Ab kit (Intec Products Inc., Xiamen, China) following the manufacturer’s instructions. Serum was detected for the presence of hepatitis B surface antigen (HBsAg) using ELISA kits (Beijing Wantai Biological Pharmacy Enterprise Co., Ltd., Beijing, China; Zhu Hai Livzon Diagnostics Inc., Zhuhai, China). Similarly, IgG antibodies of HCV were also detected by ELISA technique (Beijing Wantai Biological Pharmacy Enterprise Co., Ltd., Beijing, China; Zhu Hai Livzon Diagnostics Inc., Zhuhai, China) according to the instructions of manufacturer. Serum from all healthy blood donors was detected for the presence of treponemal antibodies by ELISA kits (Beijing Wantai Biological Pharmacy Enterprise Co., Ltd., Beijing, China; Intec Products Inc., Xiamen, China) following the manufacturer’s instructions.

### Statistical analysis

The data validated and analyzed using Statistical Package for Social Science (SPSS) for Windows version 17.0 (SPSS Inc., Chicago, IL, USA). The seroprevalences of HIV, HBV, HCV, and *T. pallidum* were expressed in percentages and reported with 95 % confidence intervals (95 % CI) [10] for the entire study group and by age, gender and geographical region. Differences in prevalence of TTSs for socio-demographic variables were checked for significance by logistic regression. Additionally, the variation tendency in seroprevalence of these blood-borne pathogens over the study period was evaluated by Cochran-Armitage trend test from Statistical Analysis System 9.3 (SAS Institute Inc., NC, USA). Statistical significance was defined as a *P*-value less than 0.05.

## Results

### Demographic characteristics of blood donors

As shown in Table 1, overall of 212 639 consecutive blood donors were screened. Of these, 58.1 % blood donors were males and 41.9 % were females. The middle age of the subjects was 35 years (range 18–65 years). Of all blood donors, 32.7 % were in the age group of 36–45 years, 32.3 % were blood group O (Table 1). Most donors are resident, but a quote belongs to the floating population. In addition, workers (18.1 %), students (10.7 %), farmers (9.0 %), and merchants (8.8 %) constitute a main portion (Table 1).

**Table 1:** Socio-demographic characteristics of blood donors

Characteristics	No. (%)
Gender	
Male	122 951 (58.1)
Female	88 688 (41.9)
Age group (years)	
18–25	56 355 (26.6)
26–35	53 751 (25.4)
36–45	69 094 (32.7)
> 45	32439 (15.3)
ABO Blood groups	
O Type	68 247 (32.3)
A Type	65 653 (31.0)
B Type	57 843 (27.3)
AB Type	19 896 (9.4)
Occupation	
Worker	38 376 (18.1)
Student	22 683 (10.7)
Farmer	19 016 (9.0)
Merchant	18 636 (8.8)
Office staff	14 441 (6.8)
Doctor	13 189 (6.2)
Govt. employee	12 014 (5.7)
Teacher	8 213 (3.9)
Soldier	1 574 (0.8)
Others	63 497 (30.0)

Note: No.=Number. For occupation, the others including housewife, freelancers and migrant labors

### Trend of HIV, HBV, HCV and *T. pallidum* seroprevalence

**Fig. 1:**
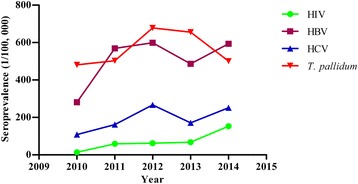
Trends of seropositivity of HIV, HBV, HCV and *Treponema pallidum* among blood donors

The overall seroprevalence of HIV, HBV, HCV and *T. pallidum* were 0.08 % (159/212 639), 0.51 % (1 087/212 639), 0.20 % (414/212 639) and 0.57 % (1 198/212 639), respectively (Table 2). Considerably increasing trends of HIV (Z = −6.88, *P* < 0.01), HBV (Z = −4.52, *P* < 0.01), HCV (Z = −4.16, *P* < 0.01) and *T. pallidum* (Z = −1.36, *P* < 0.01) seroprevalence were observed over the survey schedule (Fig. 1). The HIV seroprevalence was maintained at 0.01 % in 2010 and increased to 0.15 % in 2014. The seroprevalence of HBV was increased from 0.28 % in 2010 to 0.60 % in 2012 but later reduced to 0.49 % in 2013 and faintly increased to 0.60 % in 2014. Similar to trend of HBV, HCV prevalence increased steadily from 0.11 % in 2010 to 0.16 % in 2011, 0.27 % in 2012 but subsequently decreased to 0.17 % in 2013, and increased further to 0.25 % in 2014. On the contrary, the prevalence of *T. pallidum* increased progressively from 0.48 % in 2010 to 0.50 % in 2011, 0.68 % in 2012 and then decreased into 0.66 % in 2013 and 0.50 % in 2014 (Fig. 1 and Table 2).

**Table 2:** Seropositivity of HIV, HBV, HCV and *Treponema pallidum* among blood donors

Year	Total screened No.	HIV No. (%, 95 % CI)	HBV No. (%, 95 % CI)	HCV No. (%, 95 % CI)	*T. pallidum* No. (%, 95 % CI)
2010	35 981	5 (0.01, 0–0.02)	101 (0.28, 0.23–0.33)	39 (0.11, 0.08–0.14)	173 (0.48, 0.41–0.55)
2011	40 763	24 (0.06, 0.04–0.08)	232 (0.57, 0.5–0.64)	66 (0.16, 0.12–0.2)	205 (0.50, 0.43–0.57)
2012	41 587	26 (0.06, 0.04–0.08)	249 (0.60, 0.53–0.67)	111 (0.27, 0.22–0.32)	282 (0.68, 0.6–0.76)
2013	45 617	31 (0.07, 0.05–0.09)	222 (0.49, 0.43–0.55)	78 (0.17, 0.13–0.21)	299 (0.66, 0.59–0.73)
2014	47 691	73 (0.15, 0.12–0.18)	283 (0.60, 0.53–0.67)	120 (0.25, 0.21–0.29)	239 (0.50, 0.44–0.56)
Total	211 639	159 (0.08, 0.07–0.09)	1 087 (0.51, 0.48–0.54)	414 (0.20, 0.18–0.22)	1 198 (0.57, 0.54–0.6)

Note: No., N/A, and 95 % CI represent Number, No data, and 95 % confidence interval, respectively

## Seroprevalence of HIV, HBV, HCV and *T. pallidum*

For gender, the seropositivity of HIV (OR = 1.63, *P* < 0.001) and *T. pallidum* (OR = 1.54, *P* < 0.001) were significant difference between male and female, while the seropositivity of HBV and HCV were no difference (Table 3 and Table 4).

**Table 3:** Socio–demographic characteristics of blood donors by HIV and HBV seropositivity

Characteristics	HIV No. (%, 95 % CI)	OR (95 % CI)	*P*-Values	HBV No. (%, 95 % CI)	OR (95 % CI)	*P*-Values
Gender						
Male	73 (0.06, 0.05–0.07)	1.00	N/A	627 (0.51, 0.47–0.55)	1.00	N/A
Female	86 (0.10, 0.08–0.12)	1.63 (1.20–2.23)	0.002	460 (0.52, 0.47–0.57)	1.02 (0.90–1.15)	0.782
Age group (years)						
18–25	43 (0.076, 0.05–0.1)	1.08 (0.65–1.79)	0.776	255 (0.45, 0.39–0.51)	0.72 (0.60–0.86)	0.000
26–35	37 (0.069, 0.05–0.09)	0.97 (0.58–1.63)	0.911	221 (0.41, 0.36–0.46)	0.65 (0.54–0.79)	0.000
36–45	56 (0.081, 0.06–0.1)	1.14 (0.70–1.86)	0.589	406 (0.59, 0.53–0.65)	0.93 (0.79–1.10)	0.394
>45	23 (0.071, 0.04–0.1)	1.00	N/A	205 (0.63, 0.54–0.72)	1.00	N/A
Occupation						
Worker	21 (0.05, 0.03–0.07)	1.00	N/A	191 (0.50, 0.43–0.57)	1.00	N/A
Student	22 (0.12, 0.07–0.17)	1.77 (0.98–3.23)	0.057	90 (0.40, 0.32–0.48)	0.80 (0.62–1.02)	0.075
Farmer	21 (0.11, 0.06–0.16)	2.02 (1.10–3.70)	0.020	176 (0.93, 0.79–1.07)	1.87 (1.52–2.30)	0.000
Merchant	12 (0.06, 0.02–0.1)	1.18 (0.58–2.39)	0.653	96 (0.52, 0.42–0.62)	1.04 (0.81–1.32)	0.783
Office staff	6 (0.04, 0.01–0.07)	0.76 (0.31–1.88)	0.551	61 (0.42, 0.31–0.53)	0.85 (0.64–1.13)	0.263
Doctor	12 (0.09, 0.04–0.14)	1.66 (0.82–3.38)	0.155	30 (0.23, 0.15–0.31)	0.46 (0.31–0.67)	0.000
Govt. employee	6 (0.05, 0.01–0.09)	0.91 (0.37–2.26)	0.843	52 (0.43, 0.31–0.55)	0.87 (0.64–1.18)	0.370
Teacher	6 (0.07, 0.01–0.13)	1.34 (0.54–3.31)	0.531	38 (0.46, 0.31–0.61)	0.93 (0.66–1.32)	0.680
Soldier	1 (0.06, −0.06-0.18)	1.16 (0.16–8.64)	0.587	4 (0.25, 0–0.5)	0.51 (0.19–1.37)	0.174
Others	52 (0.08, 0.06–0.1)	1.50 (0.90–2.49)	0.116	3497 (0.55, 0.49–0.61)	1.11 (0.93–1.32)	0.269

Note: No., OR, N/A and 95 % CI represent Number, Odd Rate, No data and 95 % confidence interval, respectively

**Table 4:** Socio–demographic characteristics of blood donors by HCV and *Treponema pallidum* seropositivity

Characteristics	HCV No. (%, 95 % CI)	OR (95 % CI)	*P*-Values	*T. pallidum* No. (%, 95 % CI)	OR (95 % CI)	*P*-Values
Gender						
Male	235 (0.19, 0.17–0.21)	1.00	N/A	569 (0.46, 0.42–0.5)	1.00	N/A
Female	179 (0.20, 0.17–0.23)	1.06 (0.87–1.28)	0.583	629 (0.71, 0.65–0.77)	1.54 (1.37–1.72)	0.000
Age group (years)						
18–25	132 (0.23, 0.19–0.27)	1.27 (0.93–1.72)	0.128	132 (0.23, 0.19–0.27)	0.33 (0.27–0.41)	0.000
26–35	96 (0.18, 0.14–0.22)	0.97 (0.70–1.33)	0.831	338 (0.63, 0.56–0.7)	0.89 (0.75–1.05)	0.175
36–45	126 (0.18, 0.15–0.21)	0.99 (0.73–1.34)	0.928	499 (0.72, 0.66–0.78)	1.02 (0.88–1.20)	0.775
>45	60 (0.18, 0.13–0.23)	1.00	N/A	229 (0.71, 0.62–0.8)	1.00	N/A
Occupation						
Worker	46 (0.12, 0.09–0.15)	1.00	N/A	228 (0.59, 0.51–0.67)	1.00	N/A
Student	66 (0.29, 0.22–0.36)	2.43 (1.67–3.54)	0.000	32 (0.14, 0.09–0.19)	0.24 (0.16–0.34)	0.000
Farmer	59 (0.31, 0.23–0.39)	2.59 (1.76–3.82)	0.000	191 (1.00, 0.86–1.14)	1.70 (1.40–2.06)	0.000
Merchant	38 (0.20, 0.14–0.26)	1.70 (1.11–2.62)	0.014	136 (0.73, 0.61–0.85)	1.23 (0.99–1.52)	0.056
Office staff	18 (0.12, 0.06–0.18)	1.04 (0.60–1.79)	0.888	79 (0.55, 0.43–0.67)	0.92 (0.71–1.19)	0.526
Doctor	18 (0.14, 0.08–0.2)	1.14 (0.66–1.96)	0.640	20 (0.15, 0.08–0.22)	0.25 (0.16–0.40)	0.000
Govt. employee	20 (0.17, 0.1–0.24)	1.39 (0.82–2.35)	0.218	47 (0.39, 0.28–0.5)	0.66 (0.48–0.90)	0.008
Teacher	13 (0.16, 0.07–0.25)	1.32 (0.71–2.45)	0.374	25 (0.30, 0.18–0.42)	0.51 (0.34–0.77)	0.001
Soldier	1 (0.06, −0.06–0.18)	0.53 (0.07–3.84)	0.792	3 (0.19, −0.03–0.41)	0.32 (0.10–1.0)	0.039
Others	135 (0.21, 0.17–0.25)	1.78 (1.27–2.48)	0.001	437 (0.69, 0.63–0.75)	1.16 (0.99–1.36)	0.071

Note: No., OR, N/A and 95 % CI represent Number, Odd Rate, No data and 95 % confidence interval, respectively

The seropositivity of HIV in each age group had roughly the same proportion (Table 3). For vocational distribution, the seropositivity of HIV were no differences beyond the farmer (Table 3). The HIV seroprevalence was drastically increased among farmers (OR = 2.02, *P* = 0.020) compared to workers (Table 3).

The seroprevalence of HBV was significantly increased among farmers (OR = 1.87, *P* <0.001) compared to workers (Table 3). Meanwhile, the seroprevalence of HBV was decreased among donors from 18 to 25 years group (OR = 0.72, *P* < 0.001) and 26–35 years group (OR = 0.65, *P* < 0.001) compared to the >45 years group, among Doctors (OR = 0.46, *P* <0.001) compared to workers (Table 3).

Among age groups, the seropositivity of HCV were no difference. For occupation, it was significant differences for farmers (OR = 2.59, *P* < 0.001), students (OR = 2.43, *P* < 0.001), merchants (OR = 1.70, *P* = 0.014) and others (OR = 1.78, *P* =0.001) compared to workers (Table 4).

The seroprevalence of *T. pallidum* was significantly increased among farmers (OR = 1.70, *P* <0.001) compared to workers (Table 4). Meanwhile, the seroprevalence of *T. pallidum* was decreased among donors from 18 to 25 years group (OR = 0.33, *P* < 0.001) compared to the >45 years group, among students (OR = 0.24, *P* <0.001), doctors (OR = 0.25, *P* < 0.001), Govt. employee (OR = 0.66, *P* = 0.008), Teacher (OR = 0.51, *P* = 0.001), and Soldier (OR = 0.32, *P* = 0.039) compared to workers (Table 4).

## Discussion

In study, 211 639 donors had been screened during the study period. According the socio-demographic characteristics, donors aged 18 to 45 years, workers, students, farmers, merchants and others including housewife, freelancers and migrant labors accounted for the major component. It notices that although the farmers remain a relatively small number (9.0 %), the seropositivity of TTIs is higher than other occupations. The seroprevalence of TTIs among female are higher than male. It indicates that the women are under steadily higher potential threat of TTIs than men. The department of maternal and child health care should strongly implement the national women and children development plan. It should be enhance health promotion program in community, put more emphasis on the mass screening and pay more attention to female population in order to improve their health conditions. For pregnant woman, the pregnancy testing should be performanced monthly for the first 28 weeks and then biweekly until 36 weeks, then weekly until deliver the baby. Besides the regular checkup, the real-time detection of TTIs should be executed base on the medical advice. It was observed significantly increasing trends of HIV, HBV, and HCV seroprevalence were among blood donors over the observational period. However, none of these blood donors showed the presence of two or more pathogenic markers.

Of the blood donors, 0.08 % tested positive for HIV. It is consistent with the previous survey at four Chinese blood centers from 2000 to 2010 [5]. Furthermore, the HIV seroprevalence is slightly less than Liaoning, Yancheng and a Rural Area from Shanxi [7], but somewhat more than Guangzhou and Xian [5, 6]. It is also significantly less than African countries such as Ethiopia [11], Cameroon [12], and Nigeria [13]. However, it is considerably higher than the developed countries including USA [14], Turkey [15] and Netherlands [16]. For HIV, the highest infectious burden occurred in the 36–45 years group. More females were seropositive for HIV than males. College students showed the highest prevalence (0.12 %), followed by farmers (0.11 %) and workers (0.05 %). At present, this phenomenon is a very serious situation. The occupation distribution and increased trends of HIV infection observed from the study suggested that a rise in HIV infections in the general population could create a protential risk to the blood supply. Previous survey in China showed that the proportion of HIV-positive cases related with men who have sex with men (MSM) have speedily enlarged over the study period [17]. Although several studies have been done on HIV infections in Shiyan, no positive patient associated to MSM was found [17], and this is obviously different from western countries [18]. We observed an obviously increasing trend in HIV infection among blood donors over the study period. It is consistent with the western countries [18]. It demonstrates that the country is still faced with a serious situation for AIDS prevention and control. There is no doubt that the health authorities including all levels of center for disease control (CDC) and blood stations need to consider strengthening efficient and effective measures to prevent this at-risk population (undergraduate students, farmers and workers). At present, the CDC could provide the free HIV consulting, testing and anti-viral treatment. Thus, the donor could get additional HIV testing way except the donation. There are implementing some measures for effective HIV/AIDS prevention and control. Details are as follows: (1) Actively rejects drugs; (2) Understanding and caring of AIDS patients; (3) Offer provider-initiated HIV testing and counselling (PITC) to all outpatients. Furthermore, some measures should be done: (1) Avoid unsafe injection or blood transfusion; (2) Teenagers should take the initiative to learn the knowledge of AIDS prevention, and spread knowledge and information to family and friends. Moreover, it is very necessary to introduce and develop the technique of nucleic acid testing to compress the window period of AIDS to enhance and guarantee clinical blood using security.

In China, the seroprevalence of HBV remains extraordinarily high and approximately 100 million individuals has the HBV infection [19]. Reports from different Chinese regions show the HBV seroprevalence ranges from 0.37 to 3.5 % [5–8]. Total seroprevalence in present study is lower than Guangzhou [5], Liaoning [5], Xi’an [6], the Rural Area from Shanxi [7], and western China [8], but is upper than Yancheng and Nanjing [5]. For the low HBV prevalence in donors, it might be related to the Blood donation propaganda and Limitations of detection methods. For Blood donation propaganda, the potential blood donation population are not allowed to donate blood, if they infect with pathogens which include HIV, HBV, HCV, and others. For detection methods particularly immunological method, it may underestimates the actual infection rate for HBV. Thus, the molecular diagnosis including polymerase chain reaction (PCR), real time fluorescence quantitive Polymerase Chain Reaction (FQ-PCR) and DNA chip should be introduced and will effectively ensure the quality of detection. It is noteworthy that the molecular diagnosis techniques has been widely applied for blood screening in early 2015 in Shiyan City. Compared with the seroprevalence of HBV infections from developing countries including Mexico [20], Nepal [21], South India [22], and some African countries including Ethiopia [11], Nigeria [23], and Mali [24], HBV harbors the intermediate level. In view of age distribution, notably increased in HBV seroprevalence was found in the 36–45 and ≥45 years groups and this is similar to previous reports in Xi’an [6]. However, it is in different from the reports at Northwest Ethiopia, in which higher prevalence was detected among youths [11]. Data reveals HBV seroprevalence has a significant rise and remains approximately steady level since 2011. It is noteworthy that although the HBV remain at the low level, it still need to strengthen monitoring. For effective control of HBV, specific measures include (1) Blocking mother-baby transmission; (2) To avoid iatrogenic transmission and (3) Prevent horizontal transmission. In China, there is a vaccination program against HBV and a revaccination schedule that coverage approximately 8 to 10 million newborns. And the mandatory schedule will be carried out since postnatal 24 h, 1 month and 6 months. However, the schedule also include recommended hepatitis b vaccine injection in people over 15 years old. For occupation, the farmer is the highest risk group of HBV infection and doctor is the lowest infectious population. For farmer, the possible reasons are as follows: (1) Missed diagnosis; (2) Poor eating habits and (3) Familial infection particularly vertical transmission. For doctor, the possible reasons include routine physical examination, good eating habits and reasonable administration. For students, in order to avoid HBV infection, schools should reinforce the health education.

In China, a total of 2 %–2.9 % of the population is HCV patients or infected with HCV [25]. With 18.84 % of the world population, the estimated HCV-infected population is more than those in Occident [26]. In present study, 0.20 % of all blood donors were seropositive for HCV antibodies. The seroprevalence rate is less than values ranging between 0.51 % and 12.7 % reported from other regions of China [5–8, 27]. Of the seropositive for HCV, 0.19 % was male, while 0.20 % was female. These results fall in line with historical findings of Yancheng [5] and Liaoning [5]. However, it is at variance with the previous reports from Xi’an [6], Nanjing [5] and Guangzhou [5] in which the anti-HCV positive blood donors were more widespreaded in male than female. The HCV infection found in this study is significantly lower than four Chinese regional blood centers [5], Xi’an [6] and five Chinese blood stations during 2008–2010 [27]. The 0.38 % found among blood donors in western part of Turkey [15], the 0.64 % in Nepal [21], the 0.7 % in Northwest Ethiopia [11], the 6.0 % in Osogbo, south-west Nigeria [13], and the 11.6 % prevalence reported among immigrants from Equatorial Guinea Living in Spain [28]. As a result, the general data in China particular in Shiyan indicates that the seroprevalence of HCV infection remains relatively low compared to other countries around the world [11, 13, 15, 21, 28]. Although the finding of a low prevalence of anti-HCV antibodies among apparently healthy blood donors in the study, it highlights the necessity to continuously monitoring that will guarantee the safety of blood collection and supply. For HCV control, there are also some measures should be abided. Such as strengthen the anti-HCV screening and propagation and education, pay attention to personal hygiene and do check if necessary.

As a one of STDs, syphilis can be diffused via sexual intercourse/contact, blood transfusion and vertical transmission [11]. In China, the syphilis was eliminated for two decades (1960–80) following a large scale prevention and control project [6]. However, with the pace of Chinese economic reform, several STDs including *T. pallidum* came back since the 1980s [29, 30]. It reported incidence rate of *T. pallidum* in the general population flew to 0.023 % in 2009 compared with 0.005 % in 1999 [6]. Recently, it has been reported the seroprevalence of *T. pallidum* ranged from 0.31 to 0.70 % among blood donors in different areas of China [5, 6, 8]. The previous report from India shows the different *T. pallidum* seroprevalence range from 0.19 to 0.28 % [31, 32], the difference are lower than that in provinces of China. The study shows the highest seroprevalence of *T. pallidum* among the four pathogens. It was observed that 0.57 % of the prospective blood donors had *T. pallidum* infection. In gengeral, the *T. pallidum* frequently were co-infected with HIV. However, there is no evidence showed that co-infection of *T. pallidum* and HIV in our study. It demonstrates that the prevalence of *T. pallidum* is not serious than other survey in China [33]. The seroprevalence of *T. pallidum* (0.57 %) is higher than Nanjing [5], Guangzhou [5], and Xi﻿’an [6],﻿ ﻿but is lower than Liaoning and Yancheng [5]. In sub-Saharan Africa, *T. pallidum* still remains a severe public health problem. The prevalence of *T. pallidum* infection from African countries showed 1.1 % in Osogbo, south-west Nigeria [13], 1.2 % in Mozambique [34], 2.1 % in Burkina Faso, West Africa [35], and 8.1 % in Douala, Cameroon [12]. Compared with African countries, the seroprevalence of *T. pallidum* infection in our study is significantly low. For the syphilis infection, there is no obviously increasing or decreasing trend during 2010–2014. It is clearly distinguished with dynamic syphilis infection from western developed countries [36]. However, none can afford to neglect the *T. pallidum* infection, in order to avoid a similar debacle. Thus, there is a very necessary to screen all blood donors for circulating antibodies to *T. pallidum* infection, at least as a surrogate marker. For syphilis prevention of the general population, some measures are implementing: (1) Tracking patients’ sexual partners and make the necessary treatment; (2) Checking suspected patients with serological test for syphilis. Furthermore, here are some pointers and tips should be done: (1) Pregnant women with syphilis should be given a punctual and effective treatment; (2) Pay attention to details and avoid to transmit to others. For primary and secondary school students, the health education is indispensable. Some measures should be carried out: (1) Carry on the education of standard daily action; (2) Carry on the education of safe sex; (3) Periodic physical examination. For occupation, the farmer is the highest risk group of *T. pallidum* infection and soldier is the low infectious population. Actually, the soldiers have the lower risk compared with workers. In China, only those whose exam is qualified can be allowed to join the army. Before that, they have to accept a series of strict examination particular in physical examination. In army, the soldiers need for absolute adherence to the management system. All of these condition guarantee and reduce the rish of infection for the soldiers. On the contrary, the residential sanitary condition and basic medical examination can't guarantee for farmer. Additionally, this group is lack of awareness of health consciousness, health behavior and ability of self-discipline. All these resulted in highest risk for farmer.

It's worth noting that there is no co-infections found in our study. For the absence of co-infections, we deduced that due to the low individual prevalence of HIV, HBV, HCV and syphilis, the co-infections was not likely to happen. Furthermore, it may be exist the co-infections but cannot be checked out due to the limitation of immunologic diagnosis. It is also possible that improvements in technology might have made current screening reagents more specific and reliable. Thus, we will improve the detection method in the future study.

In the study, the immunological methods were used to screen the four pathogens including HIV, HBV, HCV and *T. pallidum*. For TTIs, the foremost matter is a “window period” process, which remains an enduring threats during blood transfusion [37, 38]. From the pathogens infection to the antibody produce, it usually need to be taken 2 weeks to 3 months. During the window period, the screening of blood donors with pathogens infection will be negative. This status is commonly happened and makes the risk for clinical blood consumption. Thus, more sensitivity and specificity screening methods such as nucleic acid testing including PCR, FQ-PCR, DNA chip and loop-mediated isothermal amplification (LAMP) [39] should be adopted to help detecting pathogens earlier and thus diminish the risks related with window periods. In 2015, the blood stations at all levels in Shiyan were recommended to use nucleic acid test to replace the immunological detection system.

## Conclusions

Our study indicates a remarkably low prevalence with rapidly increasing trend of TTIs at Shiyan City, Central China. Transmission of TTIs during the serologically negative developmental window of opportunity is still sufficient to threaten blood safety. Thus, strict filtering criteria to blood donors with SOPs are highly recommended to guarantee the blood safety for clinical application. It is believed that the blood safety will be further guaranteed, the TTIs and STDs will be effectively prevented in the future based on these more sensitive assays in Shiyan city. Although the co-infection prevalence of TTIs has not been found, it still needs to be studied on a large-scale with molecular detection techniques for the improved understanding of the influence on clinical application. For effective prevention and control of TTIs and STDs, it is very necessary to organize a series of campaigns including the propaganda of medical policy, vaccination plans, pre-employment examination, free TTIs testing and so on.

## Abbreviations

AIDS: Acquired Immune Deficiency Syndrome
CDC: Center for disease control
FQ-PCR: Real time fluorescence quantitive Polymerase Chain Reaction
HBV: Hepatitis B viruses
HCV: Hepatitis C viruses
HIV: Human immunodeficiency virus
LAMP: Loop-mediated isothermal amplification
PCR: Polymerase Chain Reaction
PITC: Provider-initiated HIV testing and counselling
RH: Rhesus
SOPs: Standard Operation Procedures
STDs: Sexually transmitted diseases
SYBTC: Shiyan Blood Transfusion Center
*T. pallidum*: *Treponema pallidum*
TTIs: Transfusion-transmissible infections
WHO: World Health Organization
